# Feasibility of the imatinib stop study in the Japanese clinical setting: delightedly overcome CML expert stop TKI trial (DOMEST Trial)

**DOI:** 10.1007/s10147-018-1368-2

**Published:** 2018-11-12

**Authors:** Shin Fujisawa, Yasunori Ueda, Kensuke Usuki, Hajime Kobayashi, Eisei Kondo, Noriko Doki, Takafumi Nakao, Yoshinobu Kanda, Nobuharu Kosugi, Hiroshi Kosugi, Takashi Kumagai, Hiroshi Harada, Masato Shikami, Yasuhiro Maeda, Toru Sakura, Koiti Inokuchi, Akio Saito, Yuichiro Nawa, Masahiro Ogasawara, Junji Nishida, Takeshi Kondo, Chikashi Yoshida, Hiroyuki Kuroda, Yoko Tabe, Yoshinobu Maeda, Kenji Imajo, Kensuke Kojima, Satoshi Morita, Sho Komukai, Atsushi Kawaguchi, Junichi Sakamoto, Shinya Kimura

**Affiliations:** 1 Department of Hematology, Yokohama City University Medical Center, Yokohama, Japan; 2 Department of Hematology/Oncology, Kurashiki Central Hospital, Kurashiki, Japan; 3 Department of Hematology, NTT Medical Center, Tokyo, Japan; 4 Department of Hematology, Obihiro Kosei Hospital, Obihiro, Japan; 5 Department of General Medicine, Okayama University Graduate School of Medicine, Dentistry and Pharmaceutical Sciences, Okayama, Japan; 6 Hematology Division, Tokyo Metropolitan Cancer and Infectious Diseases Center, Komagome Hospital, Tokyo, Japan; 7 Department of Hematology, Osaka City General Hospital, Osaka, Japan; 8 Department of Hematology, Saitama Medical Center, Jichi Medical University, Saitama, Japan; 9 Department of Hematology, Numazu City Hospital, Numazu, Japan; 10 Department of Hematology, Ogaki Municipal Hospital, Ogaki, Japan; 11 Department of Hematology, Ome Municipal General Hospital, Ome, Japan; 12 Division of Hematology, Department of Medicine, Showa University Fujigaoka Hospital, Yokohama, Japan; 13 Department of Hematology, Daiyukai General Hospital, Ichinomiya, Japan; 14 Department of Hematology, National Hospital Organization Osaka Minami Medical Center, Osaka, Japan; 15 Department of Hematology, Saiseikai Maebashi Hospital, Maebashi, Japan; 16 Department of Hematology, Nippon Medical School, Tokyo, Japan; 17 Department of Hematology, Fujioka General Hospital, Fujioka, Japan; 18 Department of Hematology, Ehime Prefectural Central Hospital, Matsuyama, Japan; 19 Department of Hematology, Sapporo Hokuyu Hospital, Sapporo, Japan; 20 Division of Hematology, Saitama Medical Center, Jichi Medical University, Saitama, Japan; 21 Department of Hematology, Hokkaido University Hospital, Sapporo, Japan; 22 Department of Hematology, National Hospital Organization Mito Medical Center, Mito, Japan; 23 Department of Gastroenterology and Hematology/Clinical Oncology, Steel Muroran Memorial Hospital, Muroran, Japan; 24 Department of Next Generation Hematology Laboratory Medicine, Juntendo University Faculty of Medicine, Tokyo, Japan; 25 Department of Hematology and Oncology, Okayama University Hospital, Okayama, Japan; 26 Department of Internal Medicine, Okayama Municipal Hospital, Okayama, Japan; 27 Division of Hematology, Respiratory Medicine and Oncology, Department of Internal Medicine, Faculty of Medicine, Saga University, Saga, Japan; 28 Department of Biomedical Statistics and Bioinformatics, Kyoto University Graduate School of Medicine, Kyoto, Japan; 29 Division of Biomedical Statistics, Department of Integrated Medicine, Graduate School of Medicine, Osaka University, Osaka, Japan; 30 Division of Biomedical Statistics, Department of Integrated Medicine, Graduate School of Medicine, Osaka University, Osaka, Japan; 31 Tokai Central Hospital, Kakamigahara, Japan

**Keywords:** Chronic myelogenous leukemia, Treatment-free remission, Imatinib, Deep molecular response, Molecular recurrence-free survival

## Abstract

**Background:**

Treatment-free remission (TFR), the ability to maintain a molecular response (MR), occurs in approximately 50% of patients with chronic myelogenous leukemia (CML) treated with tyrosine kinase inhibitors (TKIs).

**Methods:**

A multicenter phase 2 trial (Delightedly Overcome CML Expert Stop TKI Trial: DOMEST Trial) was conducted to test the safety and efficacy of discontinuing imatinib. Patients with CML with a sustained MR of 4.0 or MR4.0-equivalent for at least 2 years and confirmed MR4.0 at the beginning of the study were enrolled. In the TFR phase, the international scale (IS) was regularly monitored by IS-PCR testing. Molecular recurrence was defined as the loss of MR4.0. Recurrent patients were immediately treated with dasatinib or other TKIs including imatinib.

**Results:**

Of 110 enrolled patients, 99 were evaluable. The median time from diagnosis to discontinuation of imatinib was 103 months, and the median duration of imatinib therapy was 100 months. Molecular recurrence-free survival rates were 69.6%, 68.6% and 64.3% at 6, 12, and 24 months, respectively. After discontinuation of imatinib therapy, 26 patients showed molecular recurrence, and 25 re-achieved deep MR after dasatinib treatment. Molecular response MR4.0 was achieved in 23 patients within 6 months and 25 patients within 12 months. Multivariate analysis revealed that a longer time from diagnosis to discontinuation of imatinib therapy (*p* = 0.0002) and long duration of imatinib therapy (*p* = 0.0029) predicted a favorable prognosis.

**Conclusions:**

This DOMEST Trial showed the feasibility of TKI discontinuation in a Japanese clinical setting.

## Introduction

Chronic myelogenous leukemia (CML) is a disease in which granulocytes proliferate irreversibly. The major etiology is the Philadelphia (Ph) chromosomal abnormality, which gives rise to the *BCR–ABL* fusion gene that is translated into the BCR–ABL fusion protein. CML is a hematopoietic malignancy that progresses from the chronic phase (CP) to the accelerated phase (AP), followed by the blast phase (BP). Progression from the CP, which is characterized by mild symptoms, to the BP occurs if effective treatment is not initiated. BP is a fatal condition and the median survival time without effective treatment is 3–5 years.

Imatinib mesylate is a tyrosine kinase inhibitor (TKI) that has dramatically improved the prognosis of CML and has become a standard treatment. Because of the introduction of TKIs, most CML patients can now expect long-term survival and good quality of life [1]. However, imatinib does not eradicate CML stem cells, and patients with CML are, therefore, expected to continue TKI treatment indefinitely [2]. Because of this, CML is no longer a fatal disease in most cases; however, the high cost of long-term treatment for patients with CML remains a problem. In the Stop Imatinib (STIM) study reported in 2010, approximately 40% of CML patients who achieved deep molecular response (DMR) for more than 2 years were able to safely stop imatinib [3, 4]. Several studies reported that approximately one-half of patients with CML around the world, including Japan, who maintain a molecular response (MR) to TKIs can achieve treatment-free remission (TFR) [5–9]. Furthermore, the stop second-generation (2G)-TKI multicenter observational study investigated the discontinuation of 2G-TKIs (dasatinib or nilotinib) in CML and found an earlier response than that to imatinib [10, 11].

In Japan, real-time quantitative polymerase chain reaction (RQ-PCR) analysis of BCR–ABL was not covered by the National Insurance until April 2015. As a result, TFR after imatinib treatment has not been investigated in detail in Japan. Here, we conducted a multicenter phase 2 trial (Delightedly Overcome CML Expert Stop TKI Trial: DOMEST Trial) to test the safety and efficacy of discontinuing imatinib after at least 2 years of MR4.0-equivalent sustained MR (UMIN Clinical Trials Registry UMIN000012472).

## Patients and methods

### Study design and patients

The DOMEST Trial is a phase 2, multicenter, single-arm study performed in Japan. Patients were enrolled if they had CML-CP with a sustained MR of 4.0 or MR4.0-equivalent [negative results of nested reverse transcriptase PCR (RT-PCR) assay or a highly sensitive transcription-mediated amplification method assay] for at least 2 years and confirmed MR4.0 by RQ-PCR at the beginning of the study. In the TFR phase after stopping imatinib, BCR–ABL international scale (IS)% was regularly monitored by IS-PCR testing every month for the first year and every 3 months during the second year. When molecular recurrence was detected, patients were immediately treated with dasatinib or other TKIs (including imatinib, if the patients wished). The rate of molecular recurrence was assessed in patients with at least 12 months of follow-up.

The present survey was conducted in accordance with the Declaration of Helsinki. Approval of the study protocols was obtained from the appropriate review committee or ethical committee of each institute. Written informed consent was obtained from each patient.

### Endpoints

The primary endpoint was the molecular recurrence-free survival (MRFS) rate at 6 months after discontinuation of imatinib. The secondary endpoints were the MRFS rate at 12 and 24 months after discontinuation of imatinib. Previous interferon therapy, sex, Sokal risk group, and total duration of imatinib treatment were assessed as potential prognostic factors for MRFS. The DMR rate and time to DMR of PCR-positive patients based on PCR screening during dasatinib treatment after molecular recurrence were evaluated.

### Definitions

RQ-PCR analysis was performed by Bio Medical Laboratories (BML) Inc. (Tokyo, Japan). DMR was defined as the peripheral *Major (M)-BCR–ABL*/*ABL* transcript ratio below the detection limit of the RQ-PCR analysis widely used throughout Japan [12]. Major MR (MMR) was defined as 3-log reduction (BCR–ABL^IS^ <0.1%), and MR4.0 was defined as 4-log reduction in BCR–ABL transcripts (*BCR–ABL*^*IS*^ <0.01%). Molecular recurrence was defined as loss of MR4.0 in two consecutive analyses or one loss of MR3.0 and BCR–ABL mRNA detected in two consecutive occurrences by RQ-PCR (peripheral blood) after discontinuation of imatinib treatment. Loss of MMR was defined as recurrence at that point in one measurement. MRFS was defined as the period from registration to molecular recurrence or death. Patients who died without confirmation of molecular recurrence were assumed to have progressed on the day of death. DMR with molecular recurrence after discontinuation of imatinib treatment was evaluated by BCR–ABL mRNA RQ-PCR for 18 months (1, 3, 6, 9, 12, 15, and 18 months after initiation of dasatinib). If the results were consecutively negative, the first PCR-negative day was considered the second DMR achievement date.

### Statistical analysis

The primary endpoint was estimated as the percentage of the 95% confidence interval (CI) for MRFS at 6 months after discontinuation of imatinib. The analysis determined whether the lower limit of the CI exceeded the threshold and whether the main objective of the study was achieved. The inclusion of 89 patients was required to achieve 90% power to reject the null hypothesis with a one-sided α-error of 5% if the 6-month MRFS rate was ≥ 45%, as established by Mahon et al. [3].

The number of registered cases was set at 100 patients. Previous interferon therapy, sex, Sokal risk group, and total duration of imatinib treatment were evaluated as potential prognostic factors for MRFS. The DMR rate of patients retreated with dasatinib was evaluated in patients with molecular recurrence after discontinuation of imatinib. The groups were compared using the Kaplan–Meier method and log-rank test. Univariate and multivariate Cox regression analyses were performed to identify covariates predicting longer MRFS. A stepwise multivariate approach was used to identify the most important prognostic factors with variable retention criteria for two-sided *p* < 0.05. For the multivariate model, variables showing significant relationships were used. Statistical analysis and graphical user interface were performed using statistical computing R. The cutoff data for the inclusion of data were 10/10/2017.

## Results

### Patient characteristics

**Fig. 1:**
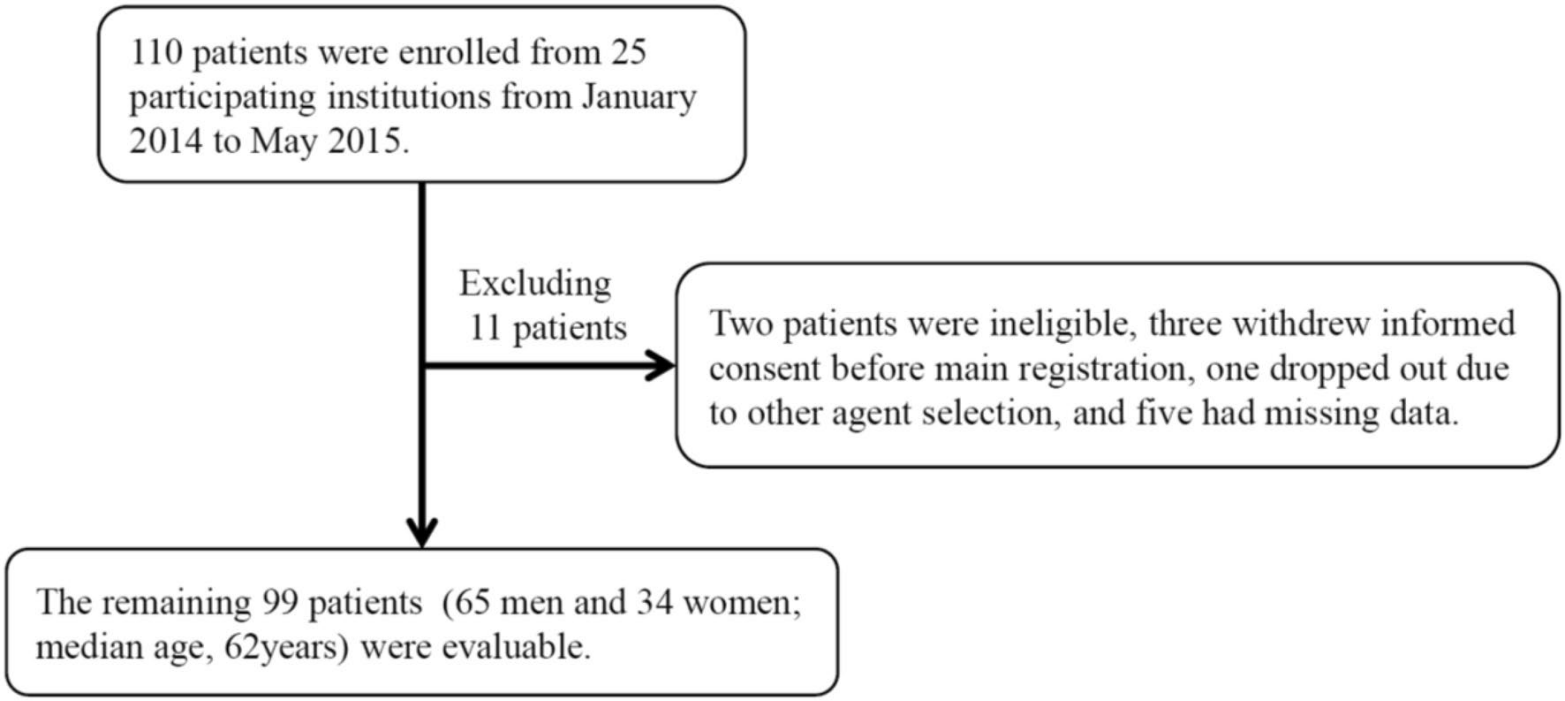
Flow diagram of patients

Patients were treated in 25 participating institutions between January 2014 and May 2015 (Fig. 1). Eleven patients were excluded (two patients were ineligible, three withdrew informed consent before main registration, one dropped out because of a different choice of treatment, and five had missing data). Of the 110 enrolled patients, 99 (65 men and 34 women with a median age of 62 years) were evaluable (Table 1). Nine patients (9%) were classified into the high-risk group according to the Sokal risk score. Sixteen patients (16%) were treated with interferon-α (IFN-α) before imatinib therapy. The median duration of IFN-α therapy was 22 (range 1–61) months, and one case was unknown. The median time from diagnosis to discontinuation of imatinib was 103 (range 29–287) months. The median duration of imatinib therapy was 100 (range 28–160) months and the MR4.0 or MR4.0-equivalent period was 55 (range 24–133) months. The median duration of DMR was 55 (range 24–133) months.

**Table 1:** Patient characteristics

Evaluable patients	n = 99
Age (years)	
Median (range)	62 years (27–88)
≤ 62	50
> 62	49
Sex	
Male	65
Female	34
Sokal score	
Low	56
Intermediate	34
High	9
Previous therapy (IFN-α)	
Yes	16
No	83
Time from diagnosis to discontinuation of imatinib (months)	
Median (range)	103 (29–287)
≤ 103	51
> 103	48
Imatinib therapy duration (months)	
Median (range)	100 (28–160)
≤ 100	51
> 100	48
Duration of DMR (months)	
Median (range)	55 (24–133)
≤ 55	50
> 55	49

*IFN-α* interferon-α, *DMR* deep molecular response

### MRFS rates at 6, 12, and 24 months

**Fig. 2:**
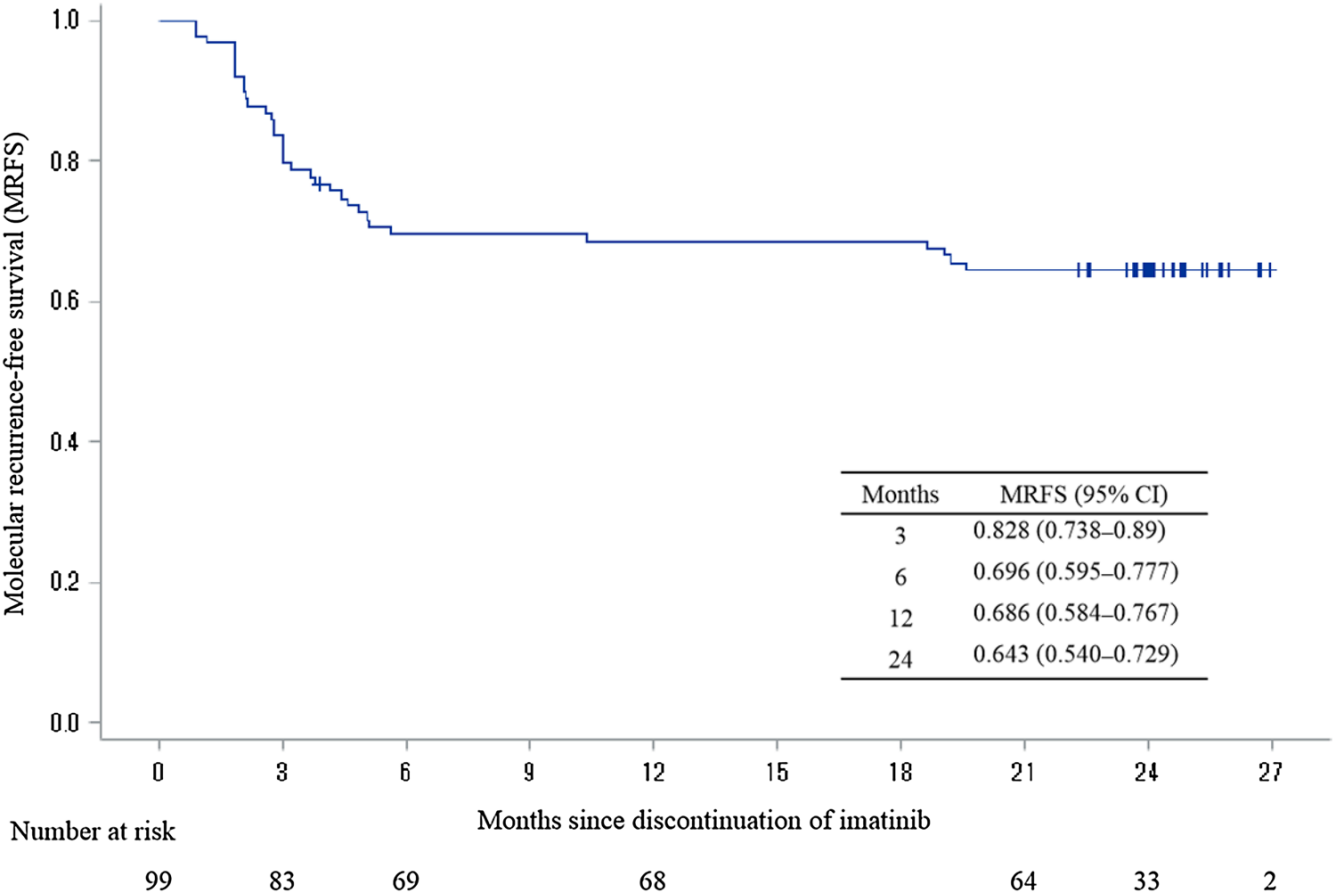
Kaplan–Meier curve of molecular recurrence-free survival (MRFS). MRFS rate at 12 months: 0.686 (95% CI 0.584–0.767)

The MRFS rates were 69.6% (95% CI 59.5–77.7%), 68.6% (95% CI 58.4–76.7%), and 64.3% (95% CI 54.0–72.9%) at 6, 12, and 24 months, respectively (Fig. 2).

### Induction of TKIs after molecular recurrence

**Fig. 3:**
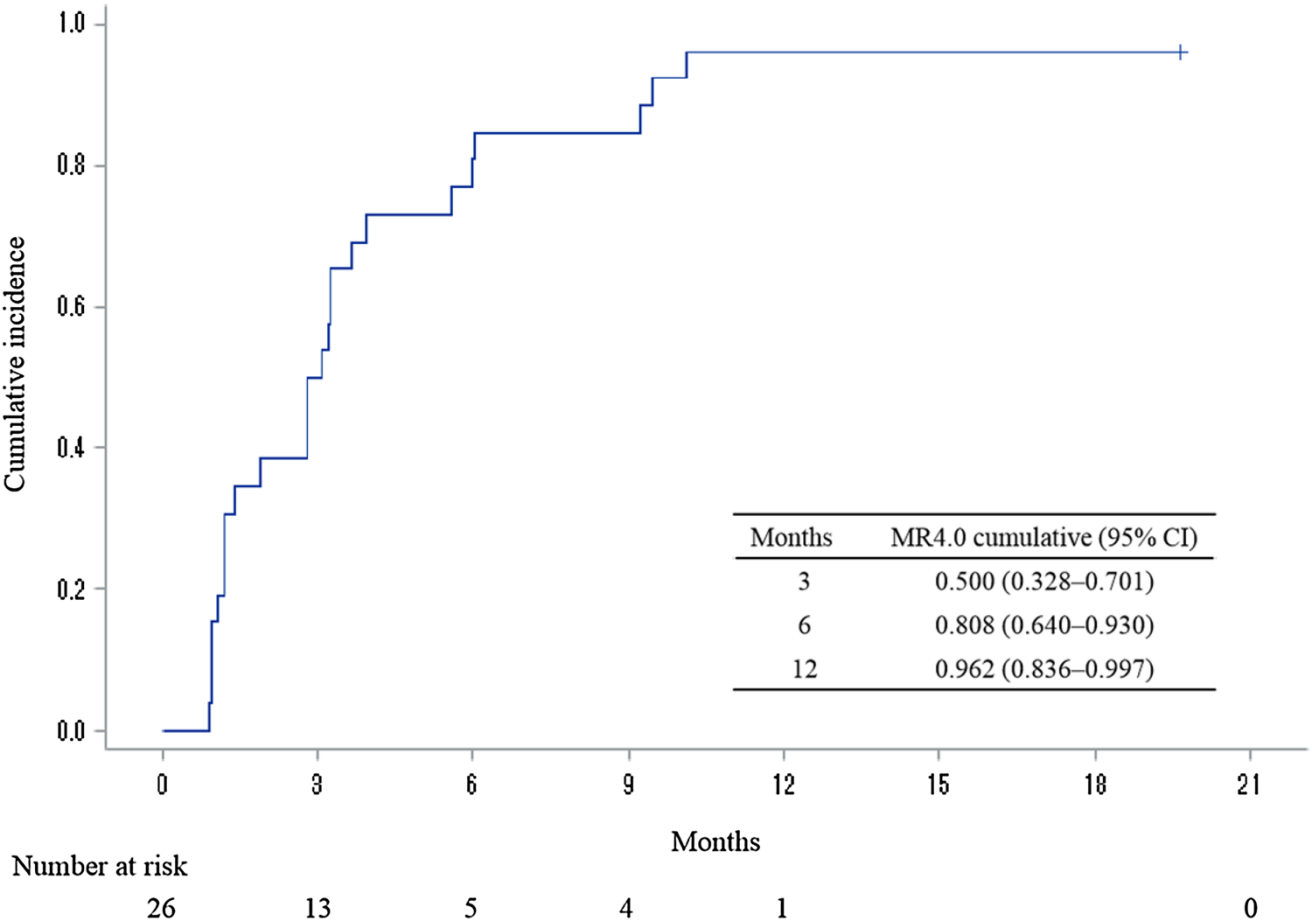
Cumulative incidence of MR4.0 re-achieved after dasatinib treatment. Twenty-one patients within 6 months and 25 within 12 months re-achieved DMR with dasatinib treatment

After discontinuation of imatinib therapy, 35 patients showed molecular recurrence, of which 26 were retreated with dasatinib and nine with imatinib. Of the 26 patients retreated with dasatinib, 21 (80.8%) re-achieved DMR within 6 months and 25 (96.2%) within 12 months. The cumulative rate of MR4.0 re-achieved after dasatinib treatment was 96.2% (95% CI 83.6–99.7%). One patient did not re-achieve MR4.0 by 18 months, but achieved MMR (Fig. 3).

### Factors associated with MRFS

**Fig. 4:**
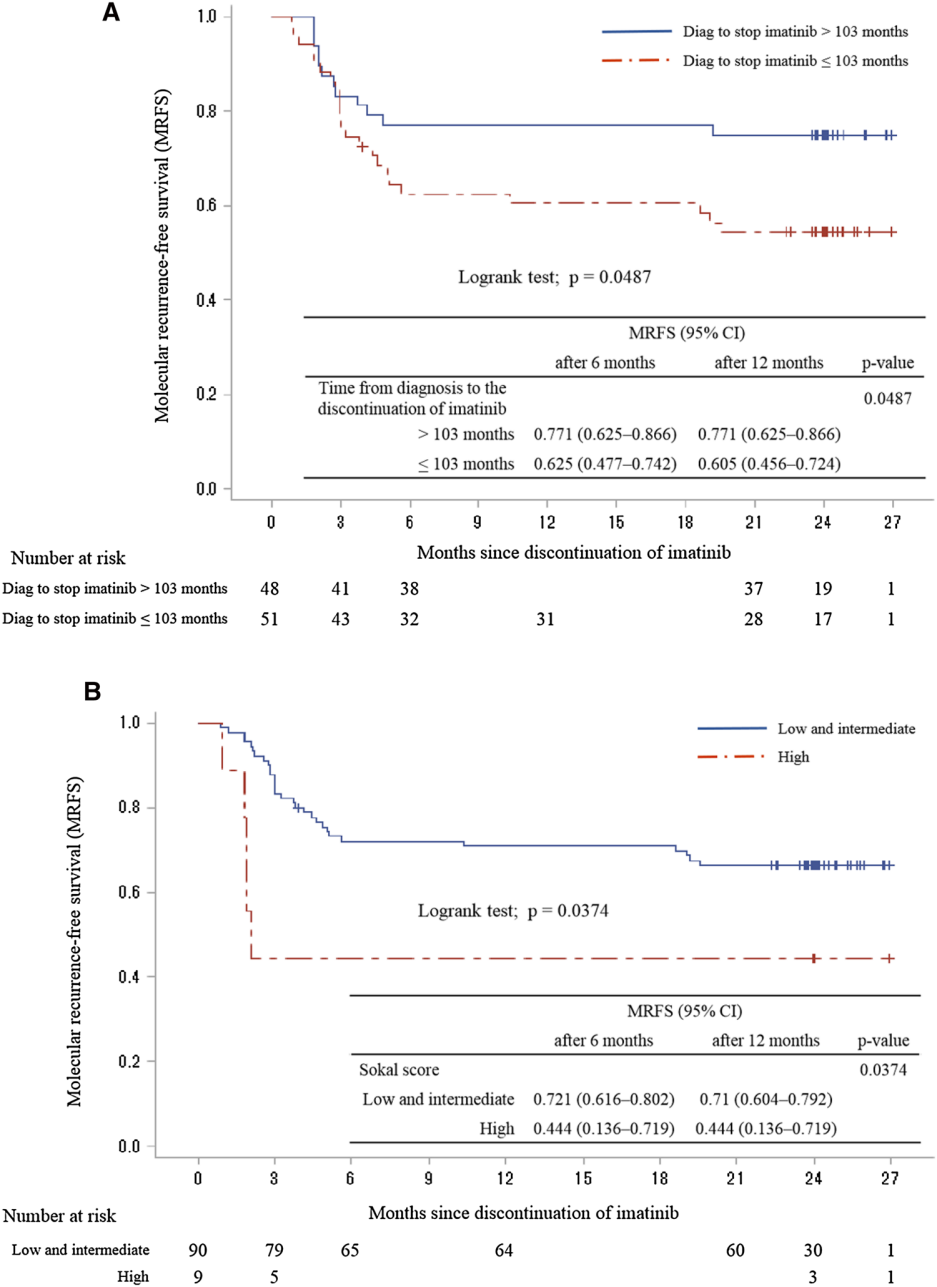
Kaplan–Meier curve of MRFS according to the time from diagnosis to the discontinuation of imatinib and Sokal score. **a** Kaplan–Meier curve of MRFS according to the time from diagnosis to the discontinuation of imatinib. MRFS rates were significantly higher in patients in which the time from diagnosis to the discontinuation of imatinib was > 103 months than in those with a shorter duration of treatment by univariate analysis (*p* = 0.0487). **b** Kaplan–Meier curve of MRFS according to Sokal score. MRFS rates were significantly higher in patients with Sokal score low or intermediate risk than in high-risk patients by univariate analysis (*p* = 0.0374)

The results of the univariate analysis of factors affecting molecular recurrence in 99 patients are shown in Table 2. The MRFS rate was significantly higher in patients with a time from diagnosis to discontinuation of imatinib > 103 months than in those with a shorter duration of treatment (*p* = 0.0487, Fig. 4-a). The MRFS rates were significantly higher in low- and intermediate-risk patients according to the Sokal score than in high-risk patients, as determined by univariate analysis (*p* = 0.0374, Fig. 4-b).

**Table 2:** Univariate analysis for molecular recurrence-free survival

	MRFS (95% CI)		*p* value
	After 6 months	After 12 months	
Age			0.5513
> 62	0.714 (0.565–0.819)	0.714 (0.565–0.819)	
≤ 62	0.68 (0.532–0.79)	0.66 (0.511–0.773)	
Sex			0.3476
Male	0.737 (0.612–0.828)	0.737 (0.612–0.828)	
Female	0.618 (0.434–0.757)	0.588 (0.406–0.732)	
Sokal score			0.0787
Low	0.713 (0.574–0.813)	0.694 (0.555–0.798)	
Intermediate	0.735 (0.553–0.853)	0.735 (0.553–0.853)	
High	0.444 (0.136–0.719)	0.444 (0.136–0.719)	
Previous therapy (IFNα)			0.4309
Yes	0.75 (0.463–0.898)	0.75 (0.463–0.898)	
No	0.685 (0.573–0.774)	0.673 (0.561–0.763)	
Time from diagnosis to the discontinuation of imatinib			0.0487
> 103	0.771 (0.625–0.866)	0.771 (0.625–0.866)	
≤ 103	0.625 (0.477–0.742)	0.605 (0.456–0.724)	
Imatinib therapy duration			0.2842
> 100	0.729 (0.58–0.833)	0.729 (0.58–0.833)	
≤ 100	0.664 (0.516–0.776)	0.644 (0.496–0.759)	
Duration of DMR			0.581
> 55	0.714 (0.566–0.82)	0.694 (0.544–0.803)	
≤ 55	0.678 (0.529–0.789)	0.678 (0.529–0.789)	

*MRFS* molecular recurrence-free survival

Multivariate analysis revealed that a longer time from diagnosis to the discontinuation of imatinib therapy and long duration of imatinib were predictive of better prognosis, whereas a high-risk classification according to the Sokal score was predictive of worse prognosis (Table 3).

**Table 3:** Multivariate analysis with the stepwise method for molecular recurrence-free survival

		HR (95% CI)	*p* value
Time from diagnosis to the discontinuation of imatinib	> 103 vs. ≤ 103	0.05 (0.01–0.25)	0.0002
Imatinib therapy duration	> 100 vs. ≤ 100	9.14 (2.14–39.08)	0.0029
Sokal risk	High vs. others	3.38 (1.27–8.99)	0.0144

*HR* hazard ratio

## Discussion

The introduction of imatinib dramatically improved the prognosis of patients with CML. In Japan, three ABL TKIs including imatinib, dasatinib and nilotinib are approved for the first-line treatment of CML. Second-generation ABL TKIs including dasatinib and nilotinib induced more rapid and deeper molecular response compared with imatinib according to DASSION and ENESTnd study [13, 14]. Based on these studies, the second-generation ABL TKIs currently tend to be chosen mainly as for the first-line treatment in Japan. However, it is also true that there is no significant difference on overall survival (OS) in patients treated either with imatinib, dasatinib or nilotinib. In point of adverse effects, it may be possible to select optimal ABL TKI for each patient. There are characteristic adverse events such as pleural effusion in dasatinib, hyperglycemia in nilotinib, and commonly cardiovascular disorders. Therefore, imatinib might be safer in patients with chest disorders, diabetes and cardiovascular events as past histories.

Stopping TKI treatment is difficult because CML stem cells cannot be eradicated, and the lifelong administration of TKIs is a problem. The possibility of TFR after discontinuation of imatinib was first evaluated in the STIM study in 100 patients with complete MR for at least 2 years [3, 4]. At the median follow-up of 65 months, the relapse-free survival was 39% at 24 months after discontinuation of imatinib. Similar findings were reported in subsequent TKI discontinuation trials [15]. Furthermore, most cases retreated with TKIs after recurrence re-achieve DMR. Therefore, patients with CML who meet the qualifying requirements and are trying to discontinue TKI treatment are considered safe [15–17].

Achieving TFR not only reduces the burden of expensive drug costs for patients, but also has a positive impact on the medical economy. In the DOMEST study, the MRFS was 69.6%, 68.6% and 64.3% at 6, 12, and 24 months, respectively (Fig. 2). However, molecular recurrence was defined as loss of MMR in the JALSG-STIM 213 study, and the results of the DOMEST study were similar to those of the JALSG-STIM 213 study [5]. In the JALSG-STIM 213 study, the median age was 55 years; 16.2% were Sokal high-risk patients, and 19.1% were treated with IFN-α before imatinib therapy. The median duration of imatinib treatment was 97.5 (range 78–130) months, and the 12-month TFR rate was 67.6%. In the present DOMEST study, the median age was slightly higher at 62 years, 9% were classified as high risk, and 16.2% were treated with IFN-α. The median duration of imatinib treatment was 100 (range 28–160) months (Table 1). Despite a slight background difference between the JALSG-STIM 213 study and the present DOMEST study, the results of the Japanese TKI discontinuation study indicate that a high TFR rate is achievable.

In previous trials, the resumption of TKI therapy immediately after recurrence resulted in the achievement of DMR in almost all patients [15]. Approximately 25–42% of patients who discontinue imatinib may experience imatinib withdrawal syndrome [3, 10, 18]. In the DOMEST study, withdrawal syndrome data were not collected, and this needs to be investigated in the future.

Studies have identified several factors for predicting recurrence after discontinuation of TKI therapy, including Sokal risk score, sex, natural killer (NK) counts, T-cell counts, suboptimal response, resistance to imatinib, duration of TKI therapy, and duration of DMR prior to TKI discontinuation [15, 19]. In the present DOMEST study, a long time period from diagnosis to the discontinuation of imatinib and long duration of imatinib therapy were predictive of better prognosis, whereas a Sokal high-risk classification was a poor predictive factor for MRFS, consistent with previous reports [3, 10]. Takahashi et al. conducted a meta-analysis of the association between the median duration of TKI and the TFR rate [5]. The duration of TKI treatment was identified as a predictive factor for TFR in previous studies [3, 4, 7, 20], and some studies reported that deeper MRs predict a greater success of TFR [8, 18]. The recent EURO-Ski study showed that the duration of DMR is more important when adjusting for the duration of TKI treatment [9]. This result may reflect early MR (EMR), because it predicts a longer duration of DMR for any given duration of TKI administration. The rates of MR and EMR are indicators of TKI sensitivity, and these were previously identified as good predictors of TFR [6, 21]. In the DOMEST study, there was no significant association between DMR duration and MRFS.

Before the introduction of TKIs, an increase of NK cells is reported in patients who discontinue IFN-α [22]. A high number of circulating NK cells and other immunological parameters are significantly correlated with improved rates of TFR [19]. In the DOMEST study, the presence of functional NK cells at the time of discontinuation of imatinib was not assessed. Dasatinib causes an increase in large granular lymphocytes (LGLs), and its therapeutic effect is high in cases with increased LGLs [23, 24]. On the basis of these results, NK cell immunity is currently considered important for the TFR of patients with CML.

The goal of CML therapy is to achieve EMR, discontinue TKIs, and maintain TFR. The cumulative 5 year MR4.5 rate in the ILIS, ENESTnd, and DASISION studies was 40.2%, 42% and 54%, respectively [13, 14, 25]. In the present DOMEST study, the 12-month TFR was 68.6%. However, if TFR is achieved by 60% of patients in the ILIS, ENESTnd, and DASISION studies, only 24.1%, 25.2%, and 32.4% of the initial CML patients can maintain TFR, respectively. To further improve the TFR rate, it is necessary to improve the DMR or the definition of recurrence, the methods of TKI discontinuation, and other variables. The results of ISAV study using digital PCR method to detect fewer minimal residual disease (MRD) in imatinib-treated CML patients showed the usefulness of the digital PCR [8]. However, the difference on the MRD levels between imatinib and second-generation ABL TKIs was not examined. In addition, it is still controversial whether deeper molecular genetic remission really increases TFR rate or not.

According to the Japanese Society of Hematology guidelines, CML patients should only discontinue imatinib therapy in the context of clinical studies [26]. However, several guidelines are presented as criteria for TKI discontinuation outside clinical trials [15–17]. Further, several patients cannot discontinue TKI therapy because there are few TKI discontinuation studies, despite the existence of patients who wish to discontinue therapy. Imatinib discontinuation could be most acceptable at present to observe the ESMO guidelines such as treatment by imatinib for more than 5 years including more than 2-year DMR [17].

The evidence from the JALSG-STIM 213 study and the present DOMEST study suggests that TKI discontinuation outside the clinical trial setting can be achieved by adapting existing guidelines.

In conclusion, the present phase 2 DOMEST study provided insight into the feasibility of stopping TKI treatment in the Japanese clinical setting, and the outcome was comparable with that of other studies investigating TFR.
